# Perceiving female physical attractiveness and expressive traits from body features and body motion

**DOI:** 10.1186/s40359-025-03522-1

**Published:** 2025-10-30

**Authors:** Lin Gao, Marc D. Pell, Zhikang Peng, Xiaoming Jiang

**Affiliations:** 1 Institute of Language Sciences and Key Laboratory of Language Science and Multilingual Intelligence Applications, Shanghai International Studies University, 1550 Wenxiang Road, 201620 Shanghai, P.R. China; 2 School of Communication Sciences and Disorders, Faculty of Medicine and Health Sciences, McGill University, 2001 McGill College, H3A1G1 Montréal, Canada

**Keywords:** Body features, Dynamic body cues, Nonverbal communication, Social perception

## Abstract

**Background:**

The perception of female physical attractiveness is known to be predicted by body features(e.g. BMI). However, the role of body motion (e.g. postures) and the relative contribution of each type of cues are unclear. Little research reported how body cues modulate the perception of female expressive traits (e.g. warmth).

**Methods:**

We photographed and filmed 15 female posers and recorded their anthropometric data. In picture stimuli, each poser adopted neutral, instructed attractive, or spontaneous attractive and unattractive postures. In video stimuli, posers introduced a place in neutral or passionate manner. Fifty-four perceivers watched these pictures and silent videos and rated their physical attractiveness and feminine expressive traits on 7-point scales.

**Results:**

Lasso regression and proportion of variance explained analyses revealed that Body features demonstrated stronger predictive power for physical attractiveness than body motions across both picture and video stimuli. However, for feminine traits, body motions showed greater predictive validity in videos, whereas neither body features nor body motions effectively predicted feminine traits in static images.

**Conclusion:**

Different roles of body features and body motion play for perceiving different levels of personal characteristics in social perception. Perception of expressive traits appears to rely more substantially on body motions, whereas the judgment of physical attractiveness depends more fundamentally on body features.

**Supplementary Information:**

The online version contains supplementary material available at 10.1186/s40359-025-03522-1.

## Introduction

 Females possessing more beautiful appearance and more positive personality traits are considered more interpersonally attractive to others [1]. Physical attractiveness, particularly related to beautiful appearance, is a crucial component of interpersonal attraction, which can influence the formation of first impressions [2, 3]. In addition to physical attractiveness, feminine expressive traits also play an important or even more pivotal role in interpersonal attraction [4]. These traits are closely associated with social attributes such as understanding, warmth, compassion, gentleness, sympathy, and sensitivity to others’ needs. Displaying more feminine expressive traits in social interaction signals one’s willingness to build or facilitate the relationship with other communicators [1].

In real-world scenarios, however, the processes involved in person perception are not entirely independent of each other. On the one hand, low-level perception (e.g., the perception of one’s physical characteristics such as attractiveness) and higher-level perception (e.g., the inference of one’s non-physical traits based on their physical characteristics) may rely on some similar cues, such as facial, vocal, and body cues. As a result, different levels of perceptual attributes can be inherently confounded in perceptual cues [5]. On the other hand, a perceiver’s perception of one level may influence another level. For example, people tend to attribute positive characteristics to physically attractive individuals [6]. Perceptually unattractive individuals, such as those who are overweight, are often stigmatized for both their appearance and personality [7]. Additionally, research has shown that the more people are familiar with, like, and respect someone, the more physically attractive they perceive that person to be [8], which further supports the notion that people’s evaluations of traits can also affect their perception of physical attractiveness.

One way to distinguish between different outcomes of person perception is to determine the perceived extent of stability. Some characteristics such as sex or race are very stable and do not easily change, which can drive prompt top-down stereotypes (Fiske & Neuberg, 1990). Compared to feminine traits, the perception of physical attractiveness may be more stable for several reasons. Firstly, physical attractiveness is influenced mainly by objective factors such as body mass [9], whereas traits are mainly influenced by subjective evaluations [10]. Secondly, the evaluation of traits depends more on different contexts, whereas physical attractiveness may depend mainly on physical characteristics and not change easily [11]. Thirdly, physical characteristics of young adults do not change sharply after adolescence (Padin, Lerner, & Spiro [12]),, whereas the evaluation of traits could easily vary over time [13]. To better understand the mechanisms underlying person perception, both perceptual cues and perception-dependent variables should be considered. Specifically, it is important to understand how different perception levels are influenced by different types of cues.

For person perception research, relevant reports have shown that static and dynamic faces convey different information in social interaction (Rubenstein, 2005), which has also been confirmed for body cues [14]. The present study sought to further explore factors that could influence the perception of physical attractiveness and feminine expressive traits. Specifically, we examined what body cues perceivers use to make judgments on the physical and feminine expressive traits, and how physical attractiveness (low-level perception) and expressive traits (high-level perception) can be distinguished based on different type of body cues.

### Interpersonal communication via body cues

The perception of body cues is an essential aspect of person perception and impression formation, which is associated with identity recognition, action perception, and mental state reasoning, and considered as a function of the ventral visual stream [15]. Prior studies have shown that body features and motions convey important information about a person’s expressions [16]. For instance, upper body features such as arms and hands can provide core features for successful emotion recognition [17], and negative body language, such as gaze fixing off centre, head stooping, rounded shoulder and narrower stance, can capture perceivers’ attention [18]. Moreover, research has shown that the motion of targets such as touch duration can influence communication [19]. Therefore, a target’s body cues should be considered as an important factor that contributes to forming interpersonal relationships.

It is important to note that body features and body motion can play distinct roles in interpersonal perception. Body features typically refer to identity-related physical features that are relatively stable, such as body shape (e.g., waist to hip ratio (WHR) [20] and body mass index (BMI) [21]. In contrast, body motion refers to transient and relatively unstable cues, such as hand gestures [22] or body postures [23]. In face-to-face social interactions, a person’s body features are typically perceived as relatively stable and static. On the other hand, body motion cues such as a speaker’s body postures and hand gestures tend to vary as communication unfolds, and even a screenshot of these cues may show a moving tendency. Given the different characteristics of these two types of body cues, we could expect them to contribute differently to low-level and high-level social perception. For instance, body features may play a role in initial social judgments related to physical attractiveness, while body motion cues could provide information about a person’s emotional state, intentions, or attitudes. Understanding the unique contributions of different body cues to social perception can help us better understand how we perceive and interact with others in different contexts.

To address this question and advance research on body perception, a more naturalistic framework which includes a larger set of body motion is needed. Body features and body motion information might not be independent [24]. Although point-light computer animation can freely manipulate static and body motion separately [24], as Johnson & Iida [25] suggested, body features and body motion cues are actually difficult to clearly isolate in real-world scenarios. More perceptual cues are also necessary to avoid limiting perceivers’ choices of perceptual cues. It would therefore be helpful to examine how person perception relies on body features and body motion using naturalistic stimuli such as videos, and more ecologically valid analysis methods which adopt a data-driven approach.

### Female physical attractiveness perception

Physical characteristics are essential for interpersonal attraction [1]. In particular, Female body cues can provide important information about their health, hormones, age and fertility [26]. These cues can serve as critical indicators of a woman’s reproductive potential, which can have essential consequences on her mating behavior and friendship selection (Tovée et al., 2001).

There have been many studies on female physical attractiveness, but most of them have focused on only a limited set of body features, such as waist-to-hip ratio (WHR) and body mass index (BMI). For instance, some studies have suggested that a WHR of 0.7 is considered the most attractive for females [20]. However, in real-life scenarios, perceivers have access to multiple body cues simultaneously, and these cues are often interdependent. Research comparing the relative contribution of waist-to-hip ratio (WHR) and body mass index (BMI) has shown that BMI could be a more important indicator of female attractiveness than WHR [27]. Furnham and colleagues [28] found that, when asked to rate line drawings of female targets varying in BMI and WHR simultaneously, perceivers rated BMI as a stronger predictor of physical attractiveness than WHR. However, focusing only on one or two body features is insufficient to explore perception in real-world scenarios. It is also unclear which body features are the strongest predictors of female physical attractiveness when multiple cues are considered together. Additionally, the relative importance of body features and body motion on female physical attractiveness remains unknown.

Some research suggests that the contribution of body features to attractiveness may differ between pictures and videos. For instance, Morrison et al. [29] used motion-capture technology to create three types of stimuli featuring female models: photographs, point-light walkers, and still frames extracted from a video. The results indicated that BMI was a stronger predictor of attractiveness in video stimuli, while WHR played an equally important role in both photographs and videos. It seems that pictures and videos are processed by different perceptual mechanisms.

Despite being largely overlooked in physical attractiveness research, body motion is likely to play a role in the perception of physical attractiveness. In a study by Röder et al. [30], male perceivers were shown dance videos of attractive and unattractive female targets, and then they were asked to rate the dancers’ attractiveness, femininity, and dance movement harmony. The study showed that male visual attention was positively associated with dance movement harmony, physically attractive dancers were perceived as more feminine and in harmony. Additionally, the posture and orientation of the body, which provide different perspectives for body observation, may also affect the perception of physical attractiveness. For instance, Rodway, Tatham, and Guo [31] found that perceivers paid more attention to front-view body than mid- and rear-view body. These findings suggest that body angle could be an important factor in perceived physical attractiveness and feminine expressive trait perception. Furthermore, as mentioned earlier, body communication may contain more information about a target’s expression, and targets have more freedom to express themselves through body motion than through body features like BMI. These details demonstrate that body motion may contribute to female physical attractiveness, although the nature of this relationship is still unclear.

### Feminine expressive traits perception

In social cognition, feminine expressive traits are associated with the warmth dimension, which includes traits related to successful social functioning (e.g., compassion, friendliness) and moral intention (e.g., honesty, trustworthiness) [32]. A target who displays more feminine expressive traits is generally perceived as being more willing to interact with others [1].

Although feminine expressive traits are important for successful social interaction, there has been little research on the relationship between the perception of body cues and feminine expressive traits. Frieze and Ramsey (1976) pointed out that due to women’s traditional role in childcare and nurturance, females tend to display more liking and warmth in nonverbal expressions than males, such as gaze and smile. Thoresen, Vuong, and Atkinson [33] used point-light techniques to record the gait of 25 walkers and asked perceivers to judge traits of targets such as approachability, extraversion, and warmth. They found that torso and limb movements were significant predictors of traits. Küster, Krumhuber, and Hess [34] also found that open posture (among other cues) influenced impressions of expressive traits such as dominance or empathic concern of female targets. Despite these previous researches, there is still a significant gap and some inconsistency in our understanding of how body cues contribute to the perception of feminine expressive traits.

### The current study

The current study investigates how perceivers utilize body features (e.g., BMI, WHR, chest circumference) and body motion (e.g., body postures, hand gestures) to judge female physical attractiveness and feminine expressive traits in social display. The primary research question is to identify the differences between the contributions of body features and body motion when perceiving female physical attractiveness or feminine expressive traits. The hypothesis is that physical attractiveness, which is built on inter-individual differences, depends more on perception of the posers’ identity-related body features, in contrast, a person’s feminine expressive traits are relatively unstable and are more likely to rely more on body motion. For instance, a female could display different levels of tenderness in various contexts and at different times through diverse forms of body motions.

## Materials and methods

### Brief summary of the materials and methods

This study utilized materials(Appendix 1) including 225 images and 60 silent videos captured by 15 female actresses. The images comprised three types of poses: 45 images in three neutral poses (frontal, left, right), 90 images in six directed attractive poses, and 90 images in six undirected poses (three with attractive postures and three without). Additionally, each actress was asked to deliver a speech about her hometown under neutral (emotionless) and passionate (emotion-filled) conditions, resulting in 30 videos for each condition. For each video, a secondary editing was performed, extracting the first 5 s after the speech began and the last 5 s before it ended for both neutral and passionate conditions, yielding a total of 60 videos capturing the actresses’ behavior in the initial and final 5 s under both conditions. After the filming, various body characteristics of each participant were recorded(such as Body Mass Index (BMI), Fluctuating Asymmetry (FA), and others as detailed in Appendix 2 Table 1313131313131313). Additionally, the body motions of the participants in the images and silent videos were coded.

A total of 54 participants in the experiment were tasked with evaluating the attractiveness and feminine expressive traits of these images and videos. The 225 images were presented in a pseudo-random order, ensuring at least four different poses between repetitions of the same pose, while the 60 videos were presented randomly. For attractiveness ratings, participants used a 1–7 Likert scale(“1” indicated extremely unattractive, and “7” indicated extremely attractive). For feminine expressive traits, participants were required to rate nine traits on a 1–7 Likert scale (“1” indicated strongly disagree, and “7” indicated strongly agree). Additionally, participants were asked to evaluate the level of passion displayed by the actresses in the videos on a scale from “1” (very unpassionate) to “7” (very passionate).

### Elicitation of stimuli

To create a perception scenario with high ecological validity, the study included both pictures and silent videos as stimuli, always showing a female actor’s whole body with their head blurred. As detailed below, for picture stimuli we further manipulated the actors’ poses (angle of view, attractive vs. unattractive), whereas for video stimuli, we manipulated the poses’ form of expression (neutral vs. passionate) and considered the role of timing in the perception of dynamic body cues (first vs. last five seconds of video) [35].

#### Posers

Fifteen young adult females (Age: 21.9 ± 1.7 years) were recruited via online university platform. Demographic information was collected online before the elicitation. All participants were physically healthy, reported to have no speech-language, neurological or psychiatric disorders, and reported to have no bruises on the skin or birthmarks or any discernable physical abnormalities. All posers provided voluntary consents and were compensated with 100 RMB for their participation.

#### Elicitation procedure

The posers were divided into two groups, with half being filmed first and the other half being photographed first. Unified clothing (tight white T-shirt and black sport shorts) was prepared for every posers. They were required to wear simple-style shoes, tied hair, and all earrings, bracelets and necklaces were removed before the experiment.

##### Filming

Posers were required to deliver speeches in a sound-proofed room. They stood in front of a white background, 4 m away from the tripod. They were filmed full body using a SONY HDR-PJ675 camera. A short speech script was sent to each poser in advance. The script was a semantically neutral introduction of their hometown. Everyone was required to memorize the script in advance. Before filming, posers were allowed to practice for a few minutes.

During elicitation, posers were asked to give a speech under two specific instructions. In the first condition, they were asked to deliver the speech with no involvement of emotional feelings, with their hands dropping naturally and faces relaxed. Their goal was to introduce their hometown in an objective way. In the second condition, posers were asked to give the speech with passion. The goal was to attract audiences to visit their hometown. Posers were encouraged to use their spontaneous body motion and gestures.

Posers were instructed to speak after hearing a “beep” sound. The duration of filming was about 15–18 s. The entire process of their speech was recorded to ensure all movements and gestures were captured. Altogether 30 videos were obtained.

##### Photographing

The same setting was applied to the photographing. Full-body pictures of each poser were shot under different postures. Three shots were taken for each pose and the one with the highest visual clarity was finally chosen. Fifteen pictures were selected for each poser, with a total of 225 pictures. There were three series:

- Neutral poses (front, left, right view): Actors stood relaxed, with arms dropping naturally. Pictures were shot from front, left and right view. Because the influence of haircut was difficult to remove, the back view of instructed postures wasn’t included. A total of 45 pictures was obtained.
- Instructed attractive poses: All posers were instructed to pose with six attractive postures shown on the computer, selected from actor catwalk or red-carpet ceremonies. No additional action demonstrations were given. A total of 90 pictures were shot.
- Spontaneous poses (spontaneous attractive and unattractive poses): “Spontaneous” means that participants received no coaching on how to appear attractive but were simply instructed to present both attractive and unattractive versions of themselves without guidance. They were encouraged to make six poses in an uninstructed, spontaneous manner. Three were poses they considered attractive and three were poses they considered unattractive. No action demonstration or verbal instructions were given. A total of 90 pictures was obtained.

##### Body Measurements

After filming and photographing, a series of body measurements were performed to collect each posers’ body features (see Appendix 2 Table 1414141414141414 for more details). All features were measured with the same standard by a female experimenter in a safe and quiet room. Each measurement was repeated twice and took the averaged value. The following measurements were conducted: Body Mass Index (BMI), the fluctuating asymmetry (FA) of body parts, chest circumference, thigh circumference, calf circumference, waist to hip ratio, finger ratio, leg-to-body ratio, head-to-body ratio, shoulder-to-hip ratio, and skin color RGB. Weight was measured on a weighing scale, with 0.1 kg error. Height was measured using a ruler with 0.5 cm error. The fluctuating asymmetry (FA) was measured using a vernier caliper, with a 0.1 cm error. Other body features were measured using a tape, with 0.1 cm error.

### Stimuli editing

#### Silent videos

 The soundtrack was first removed from all videos with Adobe Premiere pro CC. Then the first 5 s (from the onset of speech) and the last 5 s (from the offset of speech) were clipped, for both neutral and passionate videos. Each poser had 4 silent video clips (2 neutral and 2 passionate). The duration range of every poser was about 5s. Posers’ heads and faces were visually blocked with rectangular graphics tool. This procedure resulted in 60 five-second silent videos. Four conditions were created in silent videos: expression (neutral, passionate) × timing level (the first 5 s, the last 5 s).

#### Pictures

Corel Paintshop Pro (2018) was used to crop the raw pictures into 450 pixels width. The heights varied from 598 pixels to 838 pixels. Posers’ heads and faces were visually blocked by the rectangular tool in Corel Paintshop Pro (2018) and their shoes were blurred. This procedure led to 225 new photos. In pictures, three variables were involved: visual angle (left, right, front), style of attractive postures (instructed attractive poses, spontaneous attractive poses), uninstructed attractiveness (uninstructed attractive poses, spontaneous unattractive poses). Sample stimuli can be seen in the “Pictures” section of the Appendix.

### Perceptual experiment

#### Perceivers

Fifty-four new participants were recruited from Tongji university, including 27 males (Age: 22.4 ± 2.5 years old) and 27 females (Age: 21.6 ± 1.7 years old). Participants all self-reported to be single, be heterosexual, and have no diagnosed neurological or psychiatric disorders. All gave informed consent before the experiment and were compensated 80 RMB for their participation. All raters and posers shared the same cultural background.

In order to take certain perceiver-related factors into consideration, background information about perceivers was collected prior to the main experiment, including their broadcasting experience, acting experience, dancing experience, handedness, and frequency of being involved in a romantic relationship (see details reported in Supplementary Information S1). Perceivers were also required to report whether they could identify any of the other posers. None reported to recognize any posers.

#### Procedure

Perceivers completed two experimental sessions in sequence and all testing was conducted in two computer rooms each with a group of 27 perceivers. All sessions were conducted on a widely used online questionnaire platform in China (https://www.wjx.cn/). For pictures, all stimuli were pseudorandomized so that the same poses were separated by at least four other different poses. Sixty videos were randomized before being displayed for each perceiver. The procedures were as follows.

Session 1: Rating physical attractiveness and feminine expressive traits from pictures. Physical attractiveness and feminine expressive traits were judged on 225 pictures, resulting in 225 trials each requiring the participants to complete 10 ratings of the picture. The first descriptive item was “physical attractiveness (身材有吸引力)”. The other 9 items were evaluative words referring to feminine expressive traits of the actor, adapted from items of the Bem Sex Role Inventory (BSRI; Bem [2],; Geldenhuys & Bosch [3],. The Bem Sex Role Inventory (BSRI) is a commonly used tool to measure sex roles in relevant research [2]. The 9 items included: “understanding (善解人意)”, “sympathetic (为他人着想)”, “eager to soothe hurt feelings (善于安抚情绪)”, “sensitive to others’ needs (能敏感地察觉别人所需)”, “compassionate (富有同情心)”, “affectionate (关爱他人)”, “gentle (温文尔雅)”, “warm (热心)”, and “tender (温柔)”. All translations were proof-read by a graduate student who majored in English-Chinese translation.

Each trial was comprised of two sequential web pages. Each trial started with a picture which lasted for 5 s, and then it automatically turned to a second web page. On the second web page, the picture did not disappear but was zoomed out. Ten descriptive items were presented below the picture in a 7-point Likert scale. Participants were asked to rate how much they agreed on these descriptive items which were used to describe the poser (with “1” meaning strongly disagree and “7” meaning strongly agree). Participants clicked “next page” to proceed to the next trial. Three practice trials were given to get participants familiar with the procedure before the formal experiment.

Session 2: Rating physical attractiveness, feminine expressive traits and passion from silent videos. Physical attractiveness and feminine expressive traits were judged on 60 silent videos, resulting in 60 trials each requiring the participants to complete 11 ratings of one video. Participants were asked to watch a 5-second silent video by clicking the “play” button. They were allowed to watch it again if needed. In addition to the 10 descriptive items in the picture session, a new item “passionate (热情的)” was added for the participants to rate the degree of passion that the poser showed in the video.

### Data analysis

We conducted Mixed linear model to examine the impact of stimulus-related variables on ratings of physical attractiveness, feminine expressive traits, and passion (only for videos). In this section of the analysis, we considered the impact of video time sequences (first 5 s vs. last 5 s) on dynamic perceptual evaluations [35]. The reason for examining time sequences is that individuals’ behavior may change over time [36, 37] and we analyzed the first and last 5-second video clips because presentation openings and closings convey different information: openings introduce topics while closings summarize and evoke emotion [38]. A 5-second duration allows adequate evaluation of poser characteristics, as research shows humans recognize basic actions in 5–7 frames [39]. This duration was validated by eye-tracking studies finding 5 s optimal for posture perception - shorter than 3 s is insufficient, longer than 8 s induces interference [40]. Additionally, we employed Lasso regression to investigate the predictive value of body features and body motions for physical attractiveness and feminine expressive traits. These analyses encompassed both pictures and videos.

In the Lasso regression analysis, we used either body features alone, body movements alone, or their combined data to predict either physical attractiveness or feminine traits. There are three benefits to using LASSO analysis: (1) avoiding overfitting, (2) solving multicollinearity problems, and (3) conforming to the theoretical assumptions of regression analysis (i.e., all predictor variables are assumed to be fixed as a whole) [41]. The specific procedures were as follows: (1) automatically selecting the optimal regularization strength lambda through cross-validation; (2) visualizing coefficient variations across different lambda values; (3) extracting sparse coefficient matrices corresponding to critical lambda values; and (4) computing the predictive R² using the optimized model.

As a regularization method, LASSO regression introduces a penalty function to penalize overly complex models. Each variable included in the regression model generates a regression coefficient, and the sum of the absolute values of the regression coefficients is used as the penalty function to compress the regression coefficients. Lambda (0–1) is an adjustment parameter in the penalty function, which is used to control the degree of compression of the regression coefficients. As lambda approaches 1, the penalty becomes stronger, and fewer variables are included in the regression model. In this study, lambda was obtained through machine learning cross-validation using the glmnet package in R, and lambda + 1se serves as a discriminant indicator to select variables that meet the criteria and include them in the regression model. In general, we chose the lambda value that minimized the mean squared error. Given that choosing the minimum value of lambda sometimes means that the compression of the regression coefficients is relatively small and may not completely solve the problem of overfitting, some studies suggest selecting the lambda value corresponding to the standard error greater than the minimum mean squared error [42]. In all analyses, we standardized the independent variables and used the covTest package [43] to calculate *p*-values for the parameter estimates.

The Lasso regression and the construction mixed linear model were performed using R version 4.4.1.

### Body motion coding

In order to capture the variation of posers under different postures, we derived measures from other study and coded the specific body motion in silent videos and pictures [16, 18, 21].

For each silent video, the coded variables included: number of all hand gestures, number of “off-body” hand gestures (when posers kept their hands away from body sides), number of one-hand gestures, number of two-hands gestures, Time of off-body hand gestures.

For pictures, twelve variables of body motion were coded. In addition to two global styles which included posture style (instructed poses, spontaneous poses), visual angle (front, left, right), local features were also coded: leg postures (straight, curved), perceived left leg direction (how actors stretch their legs), perceived right leg position, arms (open, close, cross), arms (straight, curved), perceived left hand direction, perceived right hand direction, area of gesture (up, middle, down), perceived body tilt angle, types of postures and gestures. The detailed results of picture coding can be seen in Supplementary Information S2(Table S1: Ratio of global body features in the picture coding; Table S2-1: Ratio of local body features (hands, gestures, and body tilt angle) in the picture coding; Table S2-2: Ratio of local body features (legs and arms) in the picture coding).

## Results

### Brief summary of the results

The results comprise two main aspects: the impact of stimulus encoding variables (such as different poses, different expressions in videos, and different video timing level) on perceptual evaluations (including physical attractiveness, feminine expressive traits, and degree of passion), analyzed using Mixed linear model; and the predictive roles of body features and body movements on perceptual evaluations (including attractiveness and feminine traits), analyzed using LASSO regression.

The results of Mixed linear model indicate that, concerning different expressions in videos, evaluations of physical attractiveness, feminine traits, and passion are higher for passionate videos compared to neutral ones. Regarding different video timing levels, the last 5 s of videos exhibit more feminine traits and more passion compared to the first 5 s. Further interaction analyses reveal that this phenomenon only occurs in passionate videos, but not in neutral videos.

Concerning different poses, for neutral poses, there are no differences in perceptual evaluations among neutral poses from different perspectives. Regarding attractiveness styles (instructed attractive poses and spontaneous attractive poses), spontaneous attractive poses exhibit significantly higher evaluations in physical attractiveness and feminine traits compared to instructed attractive poses. For pose attractiveness (spontaneous attractive poses and spontaneous unattractive poses), spontaneous attractive poses receive significantly higher evaluations in physical attractiveness and feminine traits compared to spontaneous unattractive poses.

The results of Lasso regression indicate that, for silent videos, body features are more predictive of attractiveness compared to body motions, while body motions are more predictive of feminine traits compared to body features. The predictive roles of body features and body motions are stronger when combined compared to each under single conditions. For images, body features are also more predictive of attractiveness compared to body motions. However, neither body features nor body motions showed significant predictive power for feminine traits.

None of the participants reported to recognize any of the posers. Principal Component Analyses (PCA) conducted on the ratings of nine feminine expressive traits showed a single component for both silent videos and pictures. The internal reliability was found to be high in both the silent video session (Cronbach’s α = 0.958) and the picture session (Cronbach’s α = 0.943). Additionally, we further analyzed the inter-rater reliability. The results showed that the intraclass correlation coefficient (ICC) for both pictures and videos was approximately 0.5, indicating moderate consistency among raters.

### The effects of stimulus-related variables in perceptual experiment

Mixed linear model were conducted to investigate the impact of stimulus-related variables on perception in the experiment. The dependent variables were ratings of physical attractiveness, feminine expressive traits, and level of passion (only for videos), while the independent variables were stimulus-related variables. Additionally, posers’ variables (e.g., age, SPA^1^ in voice, SPA in figure and SPA in face) were included as covariates.

#### Posers’ expression and timing level in ratings of silent videos

A Mixed linear model^2^ was built to predict physical attractiveness based on posers’ expression (neutral, passionate) and timing level (first 5 s, last 5 s), with physical attractiveness as the dependent variable and subjects and actor filename as random errors. The results revealed a significant main effect of Expression(*F*(1, 3169) = 71.816, *p* < 0.001, partial *η*² =0.02), with passionate videos receiving significantly higher attractiveness ratings (*β* = 0.283, SE = 0.033, z = 8.474, *p* < 0.001). No other significant main effects or interaction effects were observed (all *ps* > 0.05).

A Mixed linear model^3^ was built to predict feminine expressive traits based on posers’ expression (neutral, passionate) and timing level (first 5 s, last 5 s), with feminine expressive traits as the dependent variable and subjects and actor filename as random errors. The analysis revealed three key findings: First, a significant main effect of Expression was observed(*F*(1, 3169) = 1266.225, *p* < 0.001, partial *η*² =0.29), with passionate videos receiving significantly higher ratings on feminine traits(*β* = 1.06, SE = 0.030, z = 35.584, *p* < 0.001). Second, Timing level showed a significant main effect (*F*(1, 3169) = 21.192, *p* < 0.001, partial *η*² =6.64e-03), where the last 5-second segments demonstrated higher feminine trait ratings compared to the first 5-second segments (*β* = 0.138, SE = 0.030, z = 4.603, *p* < 0.001). Notably, a significant interaction emerged between Expression and Timing level (*F*(1, 3169) = 10.411, *p* < 0.01, partial *η*² =3.27e-03). simple effect analysis indicated that: For passionate videos, the last 5-second segments showed significantly enhanced feminine traits compared to the first 5-second segments(*β* = 0.234, SE = 0.042, z = 5.537, *p* < 0.001), while no significant difference was found between segments in neutral videos (*β* = 0.041, SE = 0.042, z = 0.974, *p* = 0.33).

A Mixed linear model^4^ was built to predict passion based on posers’ expression (neutral, passionate) and timing level (first 5 s, last 5 s), with passion as the dependent variable and subjects and actor filename as random errors. The results showed a significant main effect of Expression (*F*(1, 3169) = 2466.007, *p* < 0.001, partial *η*² =0.44), with passionate videos receiving significantly higher passion ratings than neutral videos(*β* = 1.93, SE = 0.039, z = 49.659, *p* < 0.001). A significant main effect of Timing level was also found(*F*(1, 3169) = 17.246, *p* < 0.001, partial *η*² =5.41e-03), where the last 5-second segments had higher passion ratings compared to the first 5-second segments(*β* = 0.161, SE = 0.039, z = 4.153, *p* < 0.001). Furthermore, a significant interaction between Expression and Timing level was observed (*F*(1, 3169) = 14.220, *p* < 0.001, partial *η*² =4.47e-03): in passionate videos, the last 5-second segments showed significantly higher passion ratings than the first 5-second segments (*β* = 0.307, SE = 0.055, z = 5.603, *p* < 0.001), while no significant difference was found in neutral videos(*β* = 0.015, SE = 0.055, z = 0.270, *p* = 0.787).

#### Style of attractive postures, attractiveness in the spontaneous expression and visual angle in ratings of pictures

Further analyses investigated the impact of several variables, including the visual angle (front, left, right), style of attractive posture (instructed vs. spontaneous attractive poses) and attractiveness of spontaneous poses (spontaneous attractive poses vs. spontaneous unattractive poses), on ratings of physical attractiveness and feminine expressive traits during the picture session.

##### Visual angle in ratings of neutral poses

A Mixed linear model^5^ was built to predict physical attractiveness based on visual angle, with physical attractiveness as the dependent variable and subjects and actor filename as random errors. The results indicated that visual angle had no significant effect on physical attractiveness(*F*(2, 2360) = 0.166, *p =* 0.847, partial *η*² =1.41e-04). Similarly, we constructed a mixed linear model^6^ to predict feminine expressive traits using visual angle as the predictor, with feminine expressive traits as the dependent variable and both subjects and actor filename as random effects. The analysis revealed that visual angle did not significantly influence feminine expressive traits(*F*(2, 2360) = 2.282, *p =* 0.102, partial *η*² =1.93e-03).

##### Style of attractive posture in ratings of instructed and spontaneous poses

To further examined the predictive results of style of attractive posture on physical attractiveness, we constructed a Mixed linear model^7^ with physical attractiveness as the dependent variable and subjects and actor filename as random errors. The results revealed a significant effect of style of attractive postures on physical attractiveness(*F*(1, 7221) = 9.702, *p <* 0.01, Cohen’s *d* = −0.04), with spontaneous attractive poses demonstrating significantly higher physical attractiveness ratings compared to instructed attractive poses(*β* = 0.077, SE = 0.025, z = 3.115, *p* < 0.01). To further investigate the predictive effect of posture style on feminine expressive traits, we constructed a mixed linear model^8^, specifying feminine expressive traits as the dependent variable while including subjects and actor filename as random effects. The analysis showed a significant influence of posture style on feminine expressive traits(*F*(1, 7221) = 60.477, *p <* 0.001, Cohen’s d = −0.14), where spontaneous attractive poses received significantly higher feminine expressive trait ratings than instructed attractive poses(*β* = 0.176, SE = 0.023, z = 7.777, *p* < 0.001).

##### Attractiveness of spontaneous poses

To examined the predictive results of attractiveness in ratings of spontaneous poses on physical attractiveness, we constructed a Mixed linear model^9^ with physical attractiveness as the dependent variable and subjects and actor filename as random errors. The analysis revealed a significant effect of attractiveness of spontaneous poses on physical attractiveness(*F*(1, 4791) = 187.96, *p <* 0.001, Cohen’s *d* = 0.24). Spontaneous attractive poses received significantly higher physical attractiveness ratings compared to spontaneous unattractive poses(*β* = 0.421, SE = 0.031, z = 13.710, *p* < 0.001). To examined the predictive results of attractiveness in ratings of spontaneous poses on feminine expressive traits, we also constructed a Mixed linear model^10^ with feminine expressive traits as the dependent variable and subjects and actor filename as random errors. The results demonstrated a significant effect of attractiveness of spontaneous poses on feminine expressive traits(*F*(1, 4791) = 490.42, *p <* 0.001, Cohen’s *d* = 0.49). Spontaneous attractive poses exhibited significantly higher ratings of feminine expressive traits compared to spontaneous unattractive poses(*β* = 0.648, SE = 0.029, z = 22.145, *p* < 0.001).

### Influence of body features and body motions on attractiveness and feminine traits

The LASSO regression was employed to obtain the best fitting model, determining which variables to include in the model for optimal explanation.

#### Lasso regression of silent videos

##### Lasso regression on physical attractiveness

We conducted three models to predict physical attractiveness of silent videos based on body features and body motions.

In Model 1, we used body features to predict physical attractiveness. The LASSO regression coefficients at the optimal λ (lambda.1se) are presented in Table 1. The retained coefficients with significant *p*-values include BMI, skin color R, ring finger length, shoulder-to-hip ratio, relationship status, fluctuating Asymmetry (FA) knee breadth, SPA voice (*ps* < 0.05). We further calculated the proportion of variance explained by the model; results indicate that the body-feature-based model accounts for *R*² = 0.476 of the variance in physical attractiveness.

**Table 1:** Coefficients of retained body features from the LASSO regression

Body features	β	*p*
BMI	−1.003	< 0.0001
Skin color R	0.132	< 0.0001
Ring finger length	−0.081	< 0.0001
Shoulder-to-hip ratio	−0.148	0.0001
Relationship Status	−0.001	0.0001
FA Knee breadth	0.121	0.006
SPA voice	0.036	0.019
SPA figure	0.020	0.101
Sitting height	0.109	0.140
Menstrual period	0.040	0.545
Head height	0.044	0.917

In Model 2, we used body motions to predict physical attractiveness. The LASSO regression coefficients at the optimal λ (lambda.1se) are presented in Table 2. The retained coefficients with significant *p*-values include the numbers of double-hand gestures and numbers of off-body hand gestures (*ps* < 0.001). We further calculated the proportion of variance explained by the model; results indicate that the body-motion-based model accounts for *R*² = 0.071 of the variance in physical attractiveness.

**Table 2:** Coefficients of retained body motions from the LASSO regression

Body motions	β	*p*
Numbers of double hand gestures	0.391	< 0.0001
Time of off-body hand gestures	−0.047	< 0.0001

To investigate how the combination of static body features and dynamic movements predicts physical attractiveness, we constructed Model 3. The coefficient estimates of the LASSO regression model at the optimal lambda (lambda.1se) are shown in Table 3. The retained coefficients with significant *p*-values include BMI, shoulder-to-hip ratio, relationship status and skin color R in body features (*ps* < 0.05). In body motions, the retained coefficients with significant *p*-values include Numbers of hand gestures (*p* < 0.001). We further calculated the proportion of variance explained by the model. The results showed that the model based on body features and body motions accounts for *R*²=0.483 for physical attractiveness.

**Table 3:** Coefficients of retained body features and body motions by Lasso regression

Body features	β	*p*	Body motions	β	*p*
BMI	−0.994	< 0.0001	Numbers of hand gestures	0.095	< 0.0001
Shoulder-to-hip ratio	−0.122	0.0001	Numbers of off-body hand gestures	0.051	0.4475
Relationship Status	−0.033	0.0010			
Skin color R	0.103	0.0362			
SPA figure	0.034	0.0944			
Sitting height	0.067	0.0946			
FA Knee breadth	0.096	0.121			
Ring finger length	−0.037	0.1849			
Menstrual period	0.037	0.5103			
SPA voice	0.024	0.8280			
Head height	0.063	0.842			

Through the above analysis, we found that body features, rather than body motions, are more capable of predicting the physical attractiveness of posers in the video.

##### Lasso regression on feminine expressive traits

We conducted three models to predict feminine expressive traits if silent videos using body features and body motions.

In Model 4, we used body features to predict feminine expressive traits. The coefficient estimates of the LASSO regression model at the optimal lambda (lambda.1se) are shown in Table 4. The retained coefficients with significant *p*-values include BMI and Fluctuating asymmetry (FA) knee breadth (*ps* < 0.001). We further calculated the proportion of variance explained by the model. The results indicate that the model based on body features accounts for *R*² = 0.064 for feminine expressive traits.

**Table 4:** Coefficients of retained body features from the LASSO regression

Body features	β	*p*
BMI	−0.233	< 0.0001
FA Knee breadth	−0.653	0.0007
Acting experience	0.012	0.115

In Model 5, we used body motions to predict feminine expressive traits. The coefficient estimates of the LASSO regression model at the optimal lambda (lambda.1se) are shown in Table 5. The retained coefficients with significant *p*-values include Numbers of hand gestures and Numbers of off-body hand gestures (*ps* < 0.001). We further calculated the proportion of variance explained by the model. The results show that the model based on body motions accounts for *R*² = 0.190 for feminine expressive traits.

**Table 5:** Coefficients of retained body motions from the LASSO regression

Body motions	β	*p*
Numbers of hand gestures	0.307	< 0.0001
Numbers of off-body hand gestures	0.166	< 0.0001

To investigate how the combination of static body features and dynamic movements predicts feminine expressive traits, we constructed Model 6. The coefficient estimates of the LASSO regression model at the optimal lambda (lambda.1se) are shown in Table 6. The retained coefficients with significant *p*-values include BMI and acting experience (*ps* < 0.05). In body motions, the retained coefficients include Numbers of hand gestures and Numbers of off-body hand gestures (*ps* < 0.001). We further calculated the proportion of variance explained by the model. The results show that the model based on body features and body motions accounts for *R*²= 0.246 for feminine expressive traits.

**Table 6:** Coefficients of retained body features and body motions by Lasso regression

Body features	β	*p*	Body motions	β	*p*
BMI	−0.194	< 0.0001	Numbers of off-body hand gestures	0.276	< 0.0001
Acting experience	0.017	0.03	Numbers of hand gestures	0.269	< 0.0001
Calf circumference	−0.025	0.540			

Through the above analysis, we found that body motions, rather than body features, are more capable of predicting the feminine expressive traits of posers in the video.

#### Lasso regression of pictures

##### Lasso regression on physical attractiveness

We conducted three models to predict physical attractiveness of pictures using body features and body motions.

In Model 7, we used body features to predict physical attractiveness. The coefficient estimates of the LASSO regression model at the optimal lambda (lambda.1se) are shown in Table 7. The retained coefficients with significant *p*-values include BMI, skin color B, shoulder-to-hip ratio, chest-to-waist ratio, menstrual period and waist-to-hip ratio (*ps* < 0.05). We further calculated the proportion of variance explained by the model. The results indicate that the model based on body features accounts for *R*² = 0.403 for physical attractiveness.

**Table 7:** Coefficients of retained body features from the LASSO regression

Body features	β	*p*
BMI	−1.009	< 0.0001
Skin color B	0.012	< 0.0001
Shoulder-to-hip ratio	−0.053	0.0001
Chest-to-waist ratio	−0.063	0.0006
Menstrual period	0.118	0.003
Waist-to-hip ratio	0.027	0.021
Relationship status	−0.130	0.09
Skin color R	0.044	0.147
Finger ratio	−0.017	0.610
The most attractive aspect	0.057	0.768
Sitting height	0.030	0.936

In Model 8, we used body motions to predict physical attractiveness. The coefficient estimates of the LASSO regression model at the optimal lambda (lambda.1se) are shown in Table 8. The retained coefficients with significant *p*-values include Perceived left hand position, legs position, perceived body tilt angle and particular gestures or postures (*ps* < 0.05). We further calculated the proportion of variance explained by the model. The results show that the model based on body motions accounts for *R*² = 0.021 for physical attractiveness.

**Table 8:** Coefficients of retained body motions from the LASSO regression

Body motions	β	*p*
Perceived left hand position	0.171	< 0.0001
Legs (straight, curve)	−0.159	< 0.0001
Perceived body tilt angle	0.020	< 0.0001
Particular gestures or postures	−0.117	0.011
Visual angle	0.007	0.142
Perceived left leg position	0.048	0.255
Areas of gesture	0.020	0.345
Arms (open, close, cross)	−0.006	0.999

To investigate how the combination of static body features and dynamic movements predicts physical attractiveness, we constructed Model 9. The coefficient estimates of the LASSO regression model at the optimal lambda (lambda.1se) are shown in Table 9. The retained coefficients with significant *p*-values in body features include BMI, chest-to-waist ratio, sitting height, the most attractive aspect, menstrual period and waist-to-hip ratio (*ps* < 0.05). In body motions, the retained coefficients with significant *p*-values include Legs position(straight, curve) (*p* < 0.001). We further calculated the proportion of variance explained by the model. The results show that the model based on body features and body motions accounts for *R*²= 0.419 for physical attractiveness.

**Table 9:** Coefficients of retained body features and body motions by Lasso regression

Body features	β	*p*	Body motions	β	*p*
BMI	−1.088	< 0.0001	Legs position	−0.137	< 0.0001
Chest-to-waist ratio	−0.045	< 0.0001	Visual angle	0.069	0.342
Sitting height	0.094	0.004	Perceived body tilt angle	−0.025	0.414
The most attractive aspect	0.096	0.011	Particular gestures or postures	−0.014	0.566
Menstrual period	0.175	0.013	Perceived left hand position	0.018	0.829
Waist-to-hip ratio	0.080	0.015	Perceived right hand position	0.017	0.965
Skin color R	0.054	0.109	Arms (straight, curve)	−0.007	0.981
Relationship status	−0.188	0.371			
Forefinger	−0.052	0.465			
SPA voice	0.012	0.665			

Through the above analysis, we found that body features, rather than body motions, are more capable of predicting the physical attractiveness of posers in the images.

##### Lasso regression on feminine expressive traits

We conducted several models to predict feminine expressive traits of pictures using body features and body motions.

In Model 10, we used body features to predict feminine expressive traits. The coefficient estimates of the LASSO regression model at the optimal lambda (lambda.1se) are shown in Table 10. The retained coefficients with significant *p*-values include Skin color B, BMI and Ring finger length (*ps* < 0.05). We further calculated the proportion of variance explained by the model. The results indicate that the model based on body features accounts for *R*² = 0.054 for feminine expressive traits.

**Table 10:** Coefficients of retained body features from the LASSO regression

Body features	β	*p*
Skin color B	0.148	< 0.0001
BMI	−0.076	< 0.0001
Ring finger length	0.059	< 0.0001
Chest-to-waist ratio	−0.016	0.110
SPA face	−0.034	0.947

In Model 11, we used body motions to predict feminine expressive traits. The coefficient estimates of the LASSO regression model at the optimal lambda (lambda.1se) are shown in Table 11. The retained coefficients with significant *p*-values include Perceived right hand position, perceived left hand position and areas of gesture (*ps* < 0.001). We further calculated the proportion of variance explained by the model. The results show that the model based on body motions accounts for *R*² = 0.025 for feminine expressive traits.

**Table 11:** Coefficients of retained body motions from the LASSO regression

Body motions	β	*p*
Perceived right hand position	0.052	< 0.0001
Perceived left hand position	0.045	< 0.0001
Areas of gesture	0.035	< 0.0001
Arms (open, close, cross)	−0.061	0.166
Leg position	−0.049	0.766

To investigate how the combination of static body features and dynamic movements predicts feminine expressive traits, we constructed Model 12. The coefficient estimates of the LASSO regression model at the optimal lambda (lambda.1se) are shown in Table 12. The retained coefficients with significant *p*-values in body features include Skin color B, BMI, forefinger, ring finger length (*ps* < 0.05). In body motions, the retained coefficients with significant *p*-values include Arms (open, close, cross) and perceived right hand direction (*p* < 0.01). We further calculated the proportion of variance explained by the model. The results show that the model based on body features and body motions can explain an *R*²of 0.095 for feminine expressive traits.

**Table 12:** Coefficients of retained body features and body motions by Lasso regression

Body features	β	*p*	Body motions	β	*p*
Skin color B	0.168	< 0.0001	Arms (open, close, cross)	−0.080	< 0.0001
BMI	−0.107	< 0.0001	Perceived right hand direction	0.100	0.008
Forefinger	−0.044	0.007	Area of gesture	0.066	0.077
Ring finger length	0.104	0.010	Perceived left hand direction	0.061	0.111
Chest to waist ratio	−0.076	0.052	perceived body tilt angle	−0.066	0.377
Relationship status	−0.011	0.070	Arms (straight, curved)	−0.013	0.690
SPA face	0.007	0.075	Visual angle	0.001	0.890
Menstrual period	0.018	0.140	Leg position	−0.057	0.987
Sitting height	0.061	0.673			
Leg to body ratio	0.035	0.693			
FA Foot width	0.018	0.719			
Finger ratio	−0.020	0.742			
Head to body ratio	0.005	0.822			

Through the above analysis, we found that both body motions and body features have a very limited ability to predict the feminine expressive traits of actors in the images.

#### Proportion of variance explained

To further examine the effects of body features and body movements on physical attractiveness and feminine traits, drawing on Morrison et al.’s (2013) methodology, we calculated the proportion of variance explained by body features versus body motions in predicting either physical attractiveness or feminine traits. This analysis helps determine whether the majority of variation in perceptual judgments stems primarily from the posers’ consistent physical characteristics or from their movement patterns.

##### Silent videos

For video stimuli, we similarly treated the 15 photographed posers as between-subjects variation (reflecting stable individual differences in posers’ physical characteristics), while incorporating posers’ Expression (emotional display) and Timing level (temporal dynamics) as within-subjects variations to capture movement-based differences in bodily expressivity.

To address this, we first constructed a mixed linear model^11^ based on physical attractiveness to further clarify the proportion of variance in physical attractiveness explained by between-model individual differences (reflecting stable physical characteristics) versus within-model variations in Expression and Timing level (reflecting movement dynamics). The results revealed that: Expression and Timing level as fixed effects collectively explained only 0.6% of the variance in physical attractiveness, between-model individual differences as a random effect accounted for 50.2% of the variance and the residual variance was 49.1%. This demonstrates that stable physical characteristics, rather than body motions (captured by Expression and Timing variations), predominantly explain variations in physical attractiveness.

Next, we constructed a mixed linear model^12^ based on feminine traits. The results showed that: Expression and Timing level as fixed effects explained 16.2% of the variance in feminine traits, between-model individual differences as a random effect accounted for 10.4% of the variance and the residual variance was 73.3%. Although movement-related Expression and Timing level in videos explained only 16.2% of feminine traits, these findings may suggest that body movements (captured by Expression/Timing) better capture characteristics associated with feminine traits in video stimuli compared to body features.

##### Pictures

For the photographic stimuli, we treated the 15 photographed models as between-subject variations, which reflect stable individual differences in the models’ physical characteristics, while the 15 different posing conditions were treated as within-subject variations, capturing differences in body movement patterns exhibited through different postures.

To address this, we first constructed a mixed linear model^13^ based on physical attractiveness to further disentangle the proportion of variance explained by between-model individual differences (reflecting stable physical characteristics) versus within-model postural variations (reflecting movement differences). The results revealed that: Posture as a fixed effect explained only 0.2% of the variance in physical attractiveness; Between-model individual differences as a random effect accounted for 44.1% of the variance and the residual variance was 55.7%. This pattern demonstrates that stable physical characteristics, rather than body motions, predominantly explain variations in physical attractiveness.

Next, we constructed a mixed linear model^14^ based on feminine traits. The results showed that: as a fixed effect, model postures explained only 0.2% of feminine traits, as a random effect, between-model individual differences accounted for merely 6.9% of feminine traits and the residual variance was 92.9%. This may indicate that for static images, neither body features nor body movements can adequately capture characteristics related to feminine traits. These results are consistent with the Lasso regression analysis.

## Discussion

The aim of this study was to explore how people use body features and motion to assess physical attractiveness and feminine expressive traits, using photographs and silent videos of female posers. We also gathered multiple body cues to investigate the underlying mechanisms of social perception in a data-driven manner, taking into account various contexts (e.g., different time points, instructed versus spontaneous postures, spontaneous attractive versus unattractive postures). These different contexts can enhance the generalizability and ecological validity of the findings, extending our understanding of physical attractiveness and feminine expressive traits perception to a wider range of situations.

Our findings indicate that body cues—both body features and body motion—play a role in how people perceive physical attractiveness and feminine expressive traits in women. However, perceivers appear to rely more on body features for judging physical attractiveness and more on body motion for inferring feminine expressive traits, consistent with our hypothesis.

Regarding the effects of poser-related factors, in silent videos we found that expressions of passion (compared to neutral expressions) were rated higher in perceived physical attractiveness, feminine expressive traits, and passion level. Interestingly, although there were no differences in physical attractiveness of passionate videos, the posers’ expressions in the last five seconds were rated higher on feminine expressive traits and passion level than in the first five seconds. These findings are consistent with previous research showing the role of dynamic social cues in body perception [35], possibly because the posers displayed warmer body movements (e.g., welcoming with open arms) in the last five seconds. For the pictures, we did not find any differences in physical attractiveness or feminine expressive traits of Visual angle. However, we found that spontaneous attractive expressions (compared to spontaneous unattractive expressions) were rated higher in both physical attractiveness and feminine expressive traits. Spontaneous attractive expressions (e.g., placing hands on waist, dancing, stroking hair) were perceived as higher on physical attractiveness and expressive traits than instructed attractive postures.

### Predicting physical attractiveness from body cues

The Lasso regression results demonstrate that body features alone can effectively predict physical attractiveness in both silent videos and static images. It is noteworthy indicated that some similar variables (e.g., BMI, Skin color B) that explained the largest extent of variance in physical attractiveness ratings of both silent videos and pictures, whether body motion was added or not. Among these body cues, BMI emerged as the most robust predictor of physical attractiveness in both pictures and silent videos. Thus, while both body features and body motion can predict physical attractiveness, certain body features play a more crucial role. The perception of physical attractiveness primarily relies on inter-individual differences, such as those in body shape and weight status category measured by BMI, as opposed to body motion, which is performed by the same individual.

In contrast to earlier studies [29], our findings also support the significant impact of Waist to Hip Ratio (WHR) on ratings of attractiveness of picture. In addition, our research suggested that Body Mass Index (BMI) plays a more significant role in the perception of physical attractiveness. By examining multiple body cues, our study provided a more comprehensive understanding of physical attractiveness than some previous research, which focused on only one or two static cues [27]. Our results highlighted several important indicators of physical attractiveness, including BMI, FA Knee breadth, Ring finger, and Skin color B.

### Predicting feminine expressive traits from body cues

Using body motions such as postures and gestures in silent videos is more effective than using body features alone to predict feminine expressive traits. Our findings suggested that female’s expressive body cues such as postures and gestures can influence others’ perception of feminine expressive traits.

Our study highlights both similarities and differences between physical attractiveness and feminine expressive traits as conveyed through body features and body motion. These differences can be tentatively explained by the bottom-up utilization of body cues. Body features are stable inherent physical cues, while dynamic cues are relatively unstable behavioral displays. The former relies more on an individual level, highlighting inter-individual differences, whereas dynamic cues are based on a shared body language symbolic system and highlight intra-individual differences in various contexts. In our research, we found that physical attractiveness is primarily based on visually available and externalized body features such as BMI. Given the relative stability and accessibility of BMI in different contexts over time, a female’s physical attractiveness perception is also relatively stable and can be judged at a glance during a period of social interaction. In contrast, perception of feminine expressive traits mainly relies on body motion, which constantly changes during a period of social interaction.

### Other body cues to investigate the underlying mechanisms of social perception

In our research, we incorporated multiple body cues to investigate the underlying mechanisms of social perception in various contexts, such as different time points of the video display, instructed vs. spontaneous postures, and spontaneous attractive vs. unattractive postures. These different variations can enhance the generalizability and ecological validity of our models, enabling us to extend our understanding of physical attractiveness and feminine expressive traits perception to a wider range of contexts.

The poser’s body expression played a crucial role in influencing perceptions of physical attractiveness and feminine expressive traits. In our study, we observed that the posers’ body expressions (neutral, passionate) during a silent video introduction of their hometown had an impact on the participants’ perceptions of their physical attractiveness and feminine expressive traits. The passionate expression was perceived as more attractive and was thought to exhibit more feminine expressive traits compared to the neutral expression. Our findings are consistent with those of Raines, Hechtman & Rosenthal [44], who observed that body expressions of positive affect were more physically attractive, suggesting that expressive traits may influence physical attractiveness perceptions, in contrast to the earlier statement that physical attractiveness alters expressive trait perception.

The temporal dynamics of the respective influences of these different perceptions on each other need to be examined further. Truesdale & Pell [45] found that passionate speech contains higher maximum pitch and mean amplitude than neutral speech. Therefore, we can expect that in passionate speech of silent videos, the body motion may include more positive and expansive gestures than neutral ones that can enhance one’s feminine expressive traits and physical attractiveness. For example, in the first five seconds of passionate videos, 1/3 of actors waved their hands to greet, and in the last five seconds, 2/3 of actors opened their arms in an apparent gesture of welcome. It is worth noting that we did not observe significant differences in perceived physical attractiveness among different angle views, consistent with Tovée & Cornelissen’s [27] findings.

Additionally, we found that the spontaneity of expressions (spontaneous attractive and spontaneous unattractive) also influences the perception of physical attractiveness and feminine expressive traits. Actors’ spontaneous attractive poses were perceived as more attractive than spontaneous unattractive poses. Although physical attractiveness is predominantly assessed based on stable body features such as BMI, it appears that females are adept at effectively regulating or controlling their physical attractiveness perception through body motion. This control may stem from their knowledge and experience of nonverbal behavior and its impact on others.

Posers have a sense of how to pose charmingly based on their own body features, which may be shaped by social norms or evolutionary selection. For example, out of 45 spontaneous attractive postures, 6/45 were dancing postures, 6/45 involved stroking hair, and 10/45 involved putting hands on waist to display attractiveness. Some of these spontaneous attractive poses, such as dancing and stroking hair, are courtship behaviors [46]. In addition, the perception of physical attractiveness can be influenced by the compatibility mechanism [25]. Females are stereotypically expected to behave gently and warmly, so when their behavior goes against gender expectations, they may be regarded as unattractive (Raines et al.,1990) and less feminine. In 45 spontaneous postures, some actors folded their arms over their chest (7/45), put two hands on their waist and stood with legs apart (5/45), or pretended to check their cellphone (2/45). The compatibility mechanism also has an effect when one channel is unattractive, as the impression will be influenced by disappointment with the less attractive channel [47].

We also observed that feminine expressive traits perception is more susceptible to body motion. In passionate silent videos, there was a significant increase in feminine expressive traits in the last 5 s compared to the first 5 s, while no significant differences were found in physical attractiveness perception. This implies that physical attractiveness is relatively stable over time, while feminine expressive traits are highly influenced by body motion that unfolds over time. Additionally, we found differences in feminine expressive traits perception between instructed attractive postures and spontaneous attractive postures. These findings further support the idea that feminine expressive traits are more sensitive to body motion. These results contribute to a better understanding of how body cues impact feminine expressive traits perception.

### Limitations to body communication of physical attractiveness and feminine expressive traits in current study

Our study has several limitations. Firstly, despite having abundant body features and body motions data, the diversity of body variations may be limited due to the relatively small number of posers. With only 15 posers, each variable included only small variances which may have influenced the prediction of physical attractiveness and feminine traits. In China, a BMI < 18.5 is classified as underweight, 18.5 ≤ BMI < 24 as healthy, 24 ≤ BMI < 28 as overweight, and BMI ≥ 28 as obese [48]. According to these criteria, our stimulus set comprised 4 underweight, 9 healthy, and 2 overweight performers. More recently, however, researchers have sought to improve the sensitivity of obesity screening among Chinese university students by departing from the WHO universal threshold of BMI ≥ 28 kg/m². A large-scaled survey (*N* = 6,798) found that Chinese women should be considered obese when BMI ≥ 23.41 [49]. We acknowledge that the present study recruited only 15 performers, resulting in an unbalanced distribution across BMI categories. Besides, the current footage captured only the first and last five seconds of each presentation; future studies should strive to record the full duration of the videos.

Secondly, although the current study required recording poses of female actors in different states (such as neutral, instructed attractive poses, and spontaneous attractive and unattractive poses), these poses may still be insufficient to reflect all possible states, such as instructed unattractive poses or other poses unrelated to attractiveness, which were not included in this study. Furthermore, despite encoding a vast range of female body features and motions, those features and motions not recorded may still have some impact on the results. For instance, in a previous study, researchers used eye-tracking technology and Likert-scale ratings to have participants view 68 thirty-second animations of female gait (barefoot and in high heels). By employing structural equation modeling (SEM), they constructed a causal pathway linking gait attractiveness and femininity. The findings revealed that female walkers with lower BMI, greater lumbar curvature, pronounced backward arm swing, higher cadence, and smoother leg lines were perceived as more attractive and feminine by observers [50], breast size is also considered a significant factor influencing men’s perceptions of women’s physical attractiveness [51]. It should also be noted that the current study employed an unequal number of body-motion and body-feature variables, and this imbalance may have influenced the experimental outcomes. Meanwhile, we coded only hand gestures from the videos, as Chinese speakers tend to be more reserved in verbal communication and prefer restrained hand movements to expansive body motions [52, 53]; nevertheless, future studies should implement a more fine-grained coding scheme that captures whole-body movements.

However, current study has made efforts to record relevant body features and motions to the extent possible, and the analysis conducted around poses is within the scope of the study. Therefore, the conclusions drawn are reliable and have a certain degree of generalizability. Future research could further investigate the impact of other poses and body features and motions not covered in this study on physical attractiveness and feminine traits.

In addition, our study only focused on the perception of physical attractiveness and feminine expressive traits and did not consider other factors that may influence the perception of attractiveness, such as personality and behavioral responses. It is possible that the effect of body motion on attractiveness perception may interact with these other factors. Future studies can investigate the combined effects of body motion, personality, and behavioral outcomes in interactive tasks on the perception of attractiveness.Certainly, people’s evaluations of attractiveness are not solely limited to the body. Previous research has indicated that factors such as facial features or voice can also assist individuals in making attractiveness assessments. For instance, researchers have found self-enhancement effects and familiarity effects (friendship effects) in evaluations of facial attractiveness [54] and vocal attractiveness [55, 56]. Additionally, gender differences in attractiveness assessments have been observed [57]. These related effects and conclusions may also manifest consistently in the realm of bodily attractiveness. However, as revealed by this study, the contributions of bodily features and motions to physical attractiveness are not uniform. Furthermore, face and body may conveys different information in mate selection. For example, compared to long-term relationships, male evaluations of female body were relatively more important in short-term relationships [58]. Future research should explore the distinct roles of factors such as faces, voices, and bodies in physical attractiveness assessments, and further compare the differences in body features and motions.

Furthermore, our study only included participants from a single cultural background, the generalizability of our findings to other cultures needs further testing. Previous research has found that African American college students prefer a curvy/tight/thick body type, while thinness is seen as the standard for White women. The slim-thick ideal has long been celebrated in Latino and Black cultures, whereas it has only recently gained popularity in White mainstream culture [59]. Cultural norms and values may influence the perception of attractiveness and the importance placed on certain body motions. Future studies can include participants from diverse cultural backgrounds to explore the role of culture in the perception of attractiveness and related traits.

## Conclusion

Our study suggests that in the assessment of perceptual judgments based on body cues, perception of expressive traits appears to rely more substantially on body motions, whereas the judgment of physical attractiveness depends more fundamentally on body features. Our data highlight the importance of considering both body features and body motion in the perception of social characteristics and suggest that using both pictures and silent videos as stimuli can improve the ecological validity and generalization of results. Future research can explore how other instrumental traits, such as dominance and forcefulness, are perceived from multiple body cues.

## Supplementary Information

Supplementary Material 1

Supplementary Material 2

## Data Availability

The data and materials for all experiments are available at https://osf.io/d2tkf/.

## Footnotes

1 SPA: The participants self-perceived attractiveness in their body, face and voice.

2 Model ~ lmer(physical attractiveness ~ Expression × timing level +(1|Subject)) +(1|Actor Filename))

3 Model ~ lmer(physical attractiveness ~ Expression × timing level +(1|Subject)) +(1|Actor Filename))

4 Model ~ lmer(passion ~ Expression × timing level +(1|Subject)) +(1|Actor Filename))

5 Model ~ lmer(physical attractiveness ~ visual angle+(1|Subject)) +(1|Actor Filename))

6 Model ~ lmer(feminine expressive traits ~ visual angle+(1|Subject)) +(1|Actor Filename))

7 Model ~ lmer(physical attractiveness ~ style of attractive postures+(1|Subject)) +(1|Actor Filename))

8 Model ~ lmer(feminine expressive traits ~ style of attractive postures+(1|Subject)) +(1|Actor Filename))

9 Model ~ lmer(physical attractiveness ~ attractiveness of spontaneous poses+(1|Subject)) +(1|Actor Filename))

10 Model ~ lmer(feminine expressive traits ~ attractiveness of spontaneous poses+(1|Subject)) +(1|Actor Filename))

11 model <- lmer(physical attractiveness ~ Expression × Timing level +(1|Actor Filename)).

12 model <- lmer(femininity ~ Expression × Timing level +(1|Actor Filename)).

13 model <- lmer(physical attractiveness ~ pose style + (1|Actor Filename)).

14 model <- lmer(femininity ~ pose style + (1 | Actor Filename)).
