# Supplementary material for: Perceiving female physical attractiveness and expressive traits from body features and body motion
Source: BMC Psychol. 2025 Oct 30;13:1206. doi: 10.1186/s40359-025-03522-1 (PMC12577282; doi:10.1186/s40359-025-03522-1)
Supplement: Supplementary file 1 — Supplementary Material 1 [file 40359_2025_3522_MOESM1_ESM.docx]

**Perceiving female physical attractiveness and expressive traits from body features and body motion**

**Supplementary materials**

***S1. The results of post-experiment questionnaire***

Prior to the main experiment, all participants answered a short list of on-line questions, including their broadcasting experience, acting experience, dancing experience, handedness, and frequency of being involved in a romantic relationship. Male participants also reported the frequency of browsing pictures and videos of female body per week. Among all, 94.4％ reported no broadcasting experience, and 79.6％ of them reported no drama or dancing experience. On handedness, all of them were right-handed in writing. 88.9％ of them were right handed in brushing teeth. 83.3％ of them were right handed in throwing and 75.9％ of them were right handed in taking spoon. 29.6％ reported no previous experience of being involved in a romantic relationship, 33.3％ reported having one experience, 29.6％ reported twice experiences. The remaining reported three (1.8％) or more than five times of experience (5.6％). 7.4％ of the male participants reported having never browsed pictures or videos of female body, 25.9％ reported less than 0.5 hours per week, 37.0％ reported 0.5 to 1 hour per week, 11.1％ reported 1-3 hours per week, 14.8％ reported 3 to 5 hours per week and 3.7％ reported 5-10 hours per week.

***S2. Body movements coding in silent videos and pictures***

The body movements coding of 15 performers in pictures was shown in Table S1, Table S2-1, and Table S2-2. Table S1 showed body movements coding of global posture style. Table S2-1 and Table S2-2 were local features of performers’ postures.

**Table S1** Ratio of global body features in the picture coding

| Posture style  （n=225） | Attractive Style  （n=135） | Expression  （n=90） | Posture facing  （n=225） |
| --- | --- | --- | --- |
| Fixed pose (135/225) | Acted (90/135) | Attractive (45/90) | Back (2/225) |
| Free pose (90/225) | Spontaneous (45/135) | Unattractive (45/90) | Front (159/225) |
|  |  |  | Left (47/225) |
|  |  |  | Right (17/225) |

**Table S2-1** Ratio of local body features (hands, gestures and body tilt angle) in the picture coding

| Perceived left hand direction（n=225） | Perceived right hand direction（n=225） | Types of gesture or  posture（n=225） | Area of gestures（n=165） | Perceived body tilt angle（n=225） |
| --- | --- | --- | --- | --- |
| Nostril (2/225) | Drop naturally (83/225) | Bending down (2/225) | Top (40/165) | Lower head (6/225) |
| Drop naturally (87/225) | Thigh (21/225) | Check phone (2/225) | Down (24/165) | Tilt back (7/225) |
| Thigh (3/225) | Ears (1/225) | Dance posture (5/225) | Middle (101/165) | Tilt forward (11/225) |
| Ankle (1/225) | Heels (1/225) | Dance posture (1/225) |  | Humpback (3/225) |
| Face (4/225) | Face (3/225) | Fold arms across chests (8/225) |  | Tilt right (9/225) |
| The back of head (10/225) | Wall (1/225) | Glance back (1/225) |  | Normal (174/225) |
| Wall (3/225) | Back of body (3/225) | Grip arm (8/225) |  | Tilt left (15/225) |
| Behind the body (2/225) | Front of body (2/225) | Grip arm-back (1/225) |  |  |
| Front of body (3/225) | Right of body (1/225) | Grip hand (3/225) |  |  |
| Left of body (2/225) | Scalp (3/225) | Grip wrist (10/225) |  |  |
| Head (3/225) | Right of head (4/225) | Hand on shoulder (15/225) |  |  |
| Scalp (2/225) | Right of hip (2/225) | Hand on waists (45/225) |  |  |
| The top of head (2/225) | Knee (1/225) | Hands cover face (2/225) |  |  |
| Left of head (2/225) | Chin (2/225) | Hands forward (1/225) |  |  |
| Knee (1/225) | Chests (8/225) | Hands lifting leg (1/225) |  |  |
| Chin (18/225) | Front of chests (6/225) | Hands on cheeks (3/225) |  |  |
| Chests (8/225) | Waists (28/225) | Hands on head (1/225) |  |  |
| Front of chest (3/225) | Neck (1/225) | Locking fingers (1/225) |  |  |
| Waists (39/225) | Back of left elbow (34/225) | Nose-picking (2/225) |  |  |
| Back of right elbow (1/225) | Front of left elbow (5/225) | OK sign (1/225) |  |  |
| Front of right elbow (1/225) | Left toes (1/225) | Scratch the neck (1/225) |  |  |
| Right hand finger (2/225) | Back of left wrist (12/225) | Stroking hair (21/225) |  |  |
| Right knee (1/225) | Left hand fingers (2/225) | Stroking the chin (18/225) |  |  |
| Left ear (9/225) |  | Thumbs up (1/225) |  |  |
| Left shoulder (15/225) |  | Touching ankle (1/225) |  |  |
| Left ankle (1/225) |  | V sign (1/225) |  |  |
|  |  | Finger heart signal (1/225) |  |  |
|  |  | Victory sign (1/225) |  |  |
|  |  | Picking the toes (1/225) |  |  |
|  |  | Shooting sign (1/225) |  |  |
|  |  | Close to the wall (1/225) |  |  |
|  |  | Explore the way like Wukong (1/225) |  |  |
|  |  | Hands on pockets (1/225) |  |  |
|  |  | Fingers open (1/225) |  |  |

**Table S2-2** Ratio of local body features (legs and arms) in the picture coding

| Legs straight or curve（n=225） | Perceived Left leg direction（n=225） | Perceived right leg direction（n=225） | Arms open or close（n=225） | Arms straight or curve（n=225） |
| --- | --- | --- | --- | --- |
| Legs-straight (178/225) | Hook right leg (1/225) | Back (2/225) | Arms-open (37/225) | Arms-straight (65/225) |
| Legs-curve (1/225) | Back (1/225) | Legs apart (14/225) | Arms-cross (71/225) | Arms-curve (92/225) |
| Right leg-curve (18/225) | Legs apart (14/225) | Forward (24/225) | Arms-close to body (65/225) | Right arm-curve (24/225) |
| Left leg-curve (28/225) | Forward (5/225) | Lift back (1/225) | Right arm-open (10/225) | Left arm-curve (44/225) |
|  | Lift forward (1/225) | Lift forward (1/225) | Left arm-open (42/225) |  |
|  | Behind right leg (1/225) | Lift upward (1/225) |  |  |
|  | Front of right leg (1/225) | Right (15/225) |  |  |
|  | Natural (165/225) | Right back (1/225) |  |  |
|  | Left (17/225) | Right forward (47/225) |  |  |
|  | Left front (19/225) | Natural (118/225) |  |  |
|  |  | Front of left leg (1/225) |  |  |
