# Supplementary material for: Perceiving female physical attractiveness and expressive traits from body features and body motion
Source: BMC Psychol. 2025 Oct 30;13:1206. doi: 10.1186/s40359-025-03522-1 (PMC12577282; doi:10.1186/s40359-025-03522-1)
Supplement: Supplementary file 2 — Supplementary Material 2 [file 40359_2025_3522_MOESM2_ESM.docx]

**Appendix**

**Appendix 1 Videos and Pictures Stimuli Example**

**Videos**


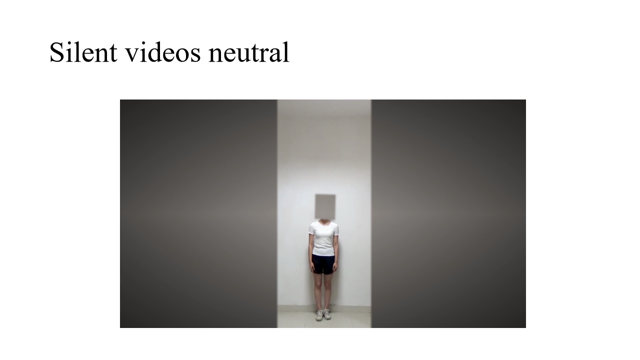


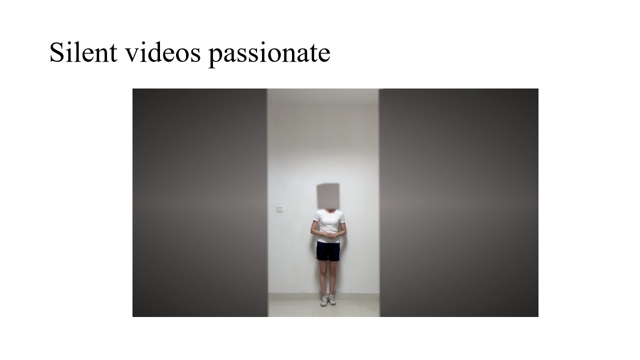


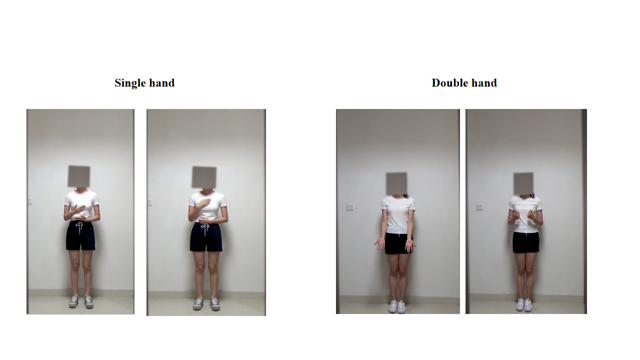


**Pictures**

**Neutral poses**


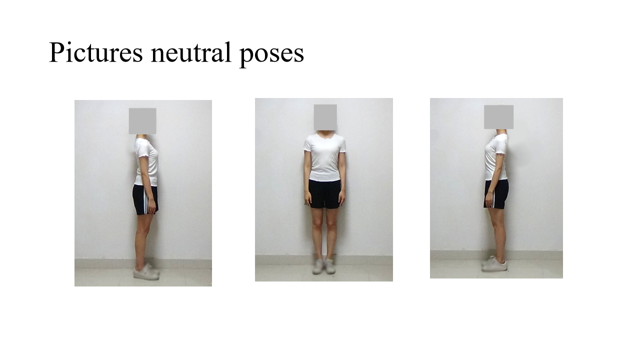


**Instructed attractive poses**


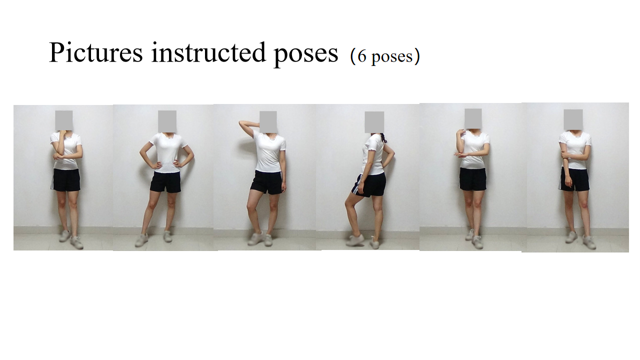


**Spontaneous attractive and unattractive poses**


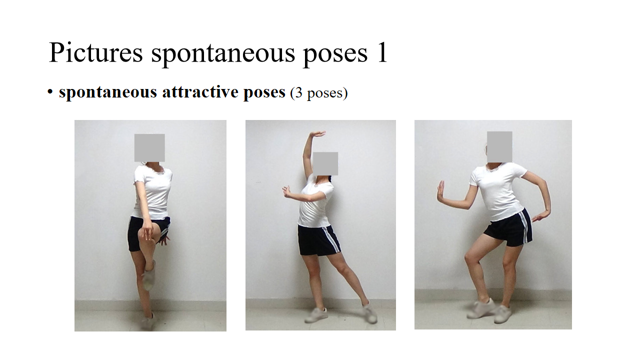


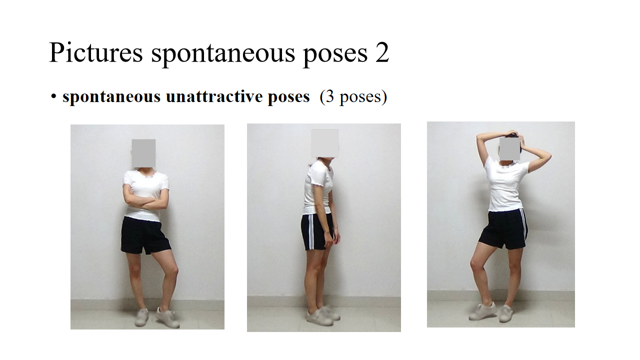


**Appendix 2**

**Table A1** The details of posers’ body measurements

| Body Measurements | Method of Measurement or Calculation |
| --- | --- |
| Body Mass Index (BMI) | Body Mass Index (BMI) was calculated from Weight (kg) and Height (m) values, with Weight as numerator, the square of Height as denominator. |
| [Fluctuating Asymmetry (FA)](file:///C:\Users\Windows\Desktop\实验修改_0720.2022\实验数据及去掉性别差异的分析\Perception_BodyAttractiveness_July202022%20-%20副本.docx#livshit) | [Fluctuating Asymmetry (FA) was measured through foot width, ankle breadth, knee breadth, elbow breadth, wrist breadth and hand breadth of the participants (Livshits & Kobyliansky, 1989). Ear length and ear breath were not measured because the face and head of models were blocked during our perceptual study.](file:///C:\Users\Windows\Desktop\实验修改_0720.2022\实验数据及去掉性别差异的分析\Perception_BodyAttractiveness_July202022%20-%20副本.docx#livshit) |
| Chest circumference | The chest circumference was measured at the breast point, and defined as the circumference at the fullest point of the bust. |
| Thigh circumference | Thigh circumference was measured horizontally around the posterior buttocks. |
| Calf circumference | The calf circumference was measured around the calf in the horizontal position and defined as the thickest part of the calf. |
| Waist-to-hip ratio (WHR) | The waist-to-hip ratio was calculated as waist circumference / hip circumference. The horizontal waist circumference was measured at the end of expiration and before the inhalation through the center of the umbilicus. The hip circumference was measured around horizontal circumference of the most prominent part of the hips backwards. |
| Finger ratio (FR) | Finger ratio was the length of the index finger divided by the length of the ring finger (from the base of the finger to the tip of the finger). |
| Leg-to-body ratio (LBR) | Leg-to-body ratio = (Height-sitting eight) / Height. |
| Head-to-body ratio (HBR) | Head-to-body ratio = height / total head height. |
| Shoulder-to-hip ratio (SHR) | Shoulder-to-hip ratio was defined as the maximum shoulder width / hip width. |
| The skin color RGB | The skin color RGB was sampled from photographs of the neutral poses in Photoshop, starting on the front of the wrist, centering on the front of the elbow joint, ending at the junction arm and short sleeves, upward for 5 points and downward for 5 points. Two points were collected on the front of the elbow. Eight points were collected from upward 1/4, 2/4, 3/4, end points and downward 1/4, 2/4, 3/4, end points. Average RGB values were calculated. |

**Table A2** Body Measurements of the 15 posers

| Performer ID | | BMI | Chest (cm) | | Thigh (cm) | Calf (cm) | WHR | FR | HBR | LBR | SHR | FA_ Foot width | FA_ Ankle breath | FA_ Knee breath | FA_ Elbow breath | FA_ Ear length | FA_ Ear breath | FA_ Wrist breath | FA_ Hand breath | Skin color_R | Skin color_G | Skin color_B |
| --- | --- | --- | --- | --- | --- | --- | --- | --- | --- | --- | --- | --- | --- | --- | --- | --- | --- | --- | --- | --- | --- | --- |
| 01 | 19.651 | | | 83 | 54.5 | 36.5 | 0.734 | 1.071 | 7.500 | 0.485 | 1.273 | 0.073 | 0.000 | 0.000 | 0.000 | 0.000 | 0.000 | 0.000 | 0.024 | 202.000 | 158.250 | 130.125 |
| 02 | 18.699 | | | 84.8 | 51.3 | 33.5 | 0.832 | 0.944 | 7.000 | 0.471 | 1.129 | 0.013 | 0.026 | 0.020 | 0.056 | 0.000 | 0.000 | 0.000 | 0.011 | 189.000 | 149.625 | 116.500 |
| 03 | 21.107 | | | 94 | 63.5 | 39 | 0.754 | 0.986 | 7.083 | 0.491 | 1.375 | 0.035 | 0.033 | 0.018 | 0.000 | 0.053 | 0.031 | 0.022 | 0.036 | 183.250 | 132.000 | 103.000 |
| 04 | 18.811 | | | 81.5 | 56.5 | 36.5 | 0.755 | 0.972 | 7.689 | 0.478 | 1.209 | 0.047 | 0.033 | 0.039 | 0.030 | 0.031 | 0.032 | 0.000 | 0.000 | 177.250 | 130.375 | 113.250 |
| 05 | 16.372 | | | 83 | 53 | 34 | 0.773 | 1.053 | 8.439 | 0.506 | 1.209 | 0.025 | 0.049 | 0.020 | 0.015 | 0.000 | 0.029 | 0.087 | 0.011 | 190.375 | 153.250 | 125.375 |
| 06 | 19.468 | | | 84 | 57.5 | 34 | 0.797 | 1.016 | 7.022 | 0.454 | 1.277 | 0.026 | 0.019 | 0.021 | 0.000 | 0.000 | 0.000 | 0.025 | 0.000 | 172.500 | 132.125 | 105.875 |
| 07 | 21.719 | | | 88.5 | 56 | 37.5 | 0.885 | 0.973 | 7.200 | 0.463 | 1.174 | 0.012 | 0.067 | 0.029 | 0.012 | 0.000 | 0.000 | 0.021 | 0.000 | 166.250 | 127.875 | 98.125 |
| 08 | 22.375 | | | 94 | 65 | 40.5 | 0.806 | 1.041 | 8.233 | 0.486 | 1.114 | 0.033 | 0.015 | 0.008 | 0.025 | 0.016 | 0.000 | 0.038 | 0.010 | 186.875 | 149.875 | 123.125 |
| 09 | 19.151 | | | 86 | 55 | 35 | 0.729 | 0.975 | 7.600 | 0.485 | 1.069 | 0.012 | 0.000 | 0.000 | 0.012 | 0.031 | 0.028 | 0.000 | 0.000 | 167.500 | 126.250 | 94.250 |
| 10 | 19.922 | | | 83 | 51 | 33 | 0.793 | 0.944 | 7.442 | 0.463 | 1.288 | 0.000 | 0.000 | 0.021 | 0.015 | 0.073 | 0.031 | 0.022 | 0.023 | 185.500 | 144.375 | 119.250 |
| 11 | 17.031 | | | 77.5 | 49.5 | 31 | 0.734 | 1.000 | 7.805 | 0.500 | 1.367 | 0.027 | 0.018 | 0.023 | 0.016 | 0.016 | 0.000 | 0.000 | 0.000 | 176.125 | 141.000 | 113.875 |
| 12 | 24.035 | | | 94.5 | 60 | 39 | 0.782 | 0.929 | 7.900 | 0.461 | 1.318 | 0.000 | 0.000 | 0.000 | 0.041 | 0.031 | 0.032 | 0.047 | 0.000 | 178.250 | 137.750 | 110.125 |
| 13 | 26.563 | | | 100.5 | 66 | 40.5 | 0.844 | 1.031 | 7.273 | 0.459 | 1.205 | 0.033 | 0.017 | 0.009 | 0.000 | 0.032 | 0.000 | 0.021 | 0.022 | 160.500 | 110.750 | 84.125 |
| 14 | 17.954 | | | 81.5 | 55 | 34 | 0.777 | 1.030 | 8.424 | 0.500 | 1.129 | 0.012 | 0.000 | 0.021 | 0.000 | 0.030 | 0.000 | 0.022 | 0.000 | 179.875 | 141.750 | 119.875 |
| 15 | 16.713 | | | 81 | 52 | 33.5 | 0.740 | 0.986 | 7.727 | 0.495 | 1.043 | 0.036 | 0.017 | 0.020 | 0.014 | 0.015 | 0.000 | 0.021 | 0.000 | 182.375 | 140.875 | 118.875 |
